# Cultural adaptation of a diabetes self-management education and support (DSMES) programme for two low resource urban settings in Ghana, during the COVID-19 era

**DOI:** 10.1186/s12913-022-08390-8

**Published:** 2022-08-05

**Authors:** Roberta Lamptey, Melanie J. Davies, Kamlesh Khunti, Sally Schreder, Bernie Stribling, Michelle Hadjiconstantinou

**Affiliations:** 1 Family Medicine and Polyclinic Department Korle, Bu Teaching Hospital, Accra, Ghana; 2 Community Health Department, University of Ghana Medical School, Accra, Ghana; 3 Julius Global Health, Julius Center for Health Sciences and Primary Care, University Medical Center Utrecht, Utrecht University, Utrecht, The Netherlands; 4 Diabetes Research Centre, Leicester Diabetes Centre, University of Leicester, Leicester, UK; 5 Leicester Diabetes Centre, University Hospitals of Leicester NHS Trust, Leicester, UK

**Keywords:** Structured diabetes self-management education, Cultural adaptation, Ghana, LMICs

## Abstract

**Background:**

Type 2 diabetes is a significant public health problem globally and associated with significant morbidity and mortality. Diabetes self-management education and support (DSMES) programmes are associated with improved psychological and clinical outcomes. There are currently no structured DSMES available in Ghana. We sought to adapt an evidence-based DSMES intervention for the Ghanaian population in collaboration with the local Ghanaian people.

**Methods:**

We used virtual engagements with UK-based DSMES trainers, produced locally culturally and linguistically appropriate content and modified the logistics needed for the delivery of the self-management programme to suit people with low literacy and low health literacy levels.

**Conclusions:**

A respectful understanding of the socio-cultural belief systems in Ghana as well as the peculiar challenges of low resources settings and low health literacy is necessary for adaptation of any DSMES programme for Ghana. We identified key cultural, linguistic, and logistic considerations to incorporate into a DSMES programme for Ghanaians, guided by the Ecological Validity Model. These insights can be used further to scale up availability of structured DSMES in Ghana and other low- middle- income countries.

## Background

The COVID-19 pandemic has heightened the need for people living with diabetes (PLWD) to be able to self-care and manage their condition more than ever before [1]. Physical restrictions and social distancing in these unprecedented times, have also highlighted the importance of not only delivering diabetes self-management education and support (DSMES) programmes but also the need to do so in a replicable and “safe” manner [2]. In Ghana, COVID-19 infections have been on the increase. As of August 2021, Ghana had 100,383 laboratory-confirmed COVID-19 virus infections and 874 COVID-19-related deaths [3, 4]. COVID-19 has disrupted clinical care in various ways. For example, there is a conscious attempt to shorten consultation time for non-urgent care, and several clinical services of Korle Bu Teaching Hospital have been shut down for varying lengths of time after COVID-19 infections staff. Additionally, the city of Accra was locked down from 30^th^ March 2021 to 19^th^ April, 2020 because of the COVID-19 pandemic. The lockdown further restricted access (physical and economic) to routine diabetes care, fresh produce and group-based outdoor physical activities e.g. street walks/jogs popularly called in local parlance ‘Djama’.

Diabetes self-management education and support (DSMES) is the foundation for effective diabetes care [5, 6]. Delivering DSMES in low resource settings and among people with low health literacy and or numeracy skills is challenging [6]; even more so during this pandemic. DSMES programmes must meet quality standards and be accredited [7]. DESMOND (Diabetes education self-management for ongoing and newly diagnosed) is a nationally accredited DSMES programme, which supports the self-management of people living with Type 2 Diabetes (T2D) [8] Originally designed in the UK, this programme is evidence- and theory-based, delivered over six hours and covers core topics relating to the pathophysiology of T2D, glycaemic control, and lifestyle management including monitoring [9]. DESMOND has been shown to improve weight loss and illness beliefs up to 12 months after diagnosis [9] and has been shown to be cost-effective [10]. In recent years, DESMOND was culturally adapted for two cities in LMICs (Lilongwe, Malawi; and Maputo, Mozambique). This adapted version, named the EXTEND programme, was tested in a feasibility trial, with results indicating positive biomedical and psychosocial improvements at both sites [11].

Usual care in public health facilities in Ghana is often based on unstructured group education prior to clinic visits. Despite the significance of DSMES programmes, evidence on the association between glycaemic control, and structured self-management education programmes in low-middle-income settings is poor [12]. In an opportunity to support a DSMES study in Ghana, collaborators from the UK and Ghana worked together to adapt the EXTEND programme and produce a programme tailored to the needs of the Ghanaian population. This paper aims to describe the adaptations to EXTEND to create a programme that is culturally and linguistically sensitive to the Ghanaian community. The objective of this manuscript is to share learnings from this adaptation process, with fellow colleagues who wish to adapt and implement DSMES in other LMICs.

## Methods

The EXTEND study was presented at an international diabetes conference in 2019 [13]. This paved the way for collaborators in Ghana and UK to begin discussions on delivering a modified version of EXTEND in Ghana. Several virtual consultative meetings were held between the two parties throughout 2020–2021. The EXTEND resources were sent to the Ghana research team by post and electronically. To ensure that the resources were culturally and linguistically appropriate to the Ghanaian population, work was conducted remotely between the UK and Ghana partners around the adaptation of the content and images of the EXTEND resources. The adaptation was led by the lead author and conducted by the Ghanaian team. This work was informed by prior extensive experience in intervention development, delivery of training and a clear understanding of the Ghanaian landscape. This work, was also informed by informal feedback from patients from routine clinics in Ghana, This feedback was in the form of handwritten notes, with the aim to capture comments and the overall experience of people living with T2D. This acted as a strong foundation of what the population’s needs and preferences are. Taking all this information and experience into consideration, we proceeded with the adaption of a self-management programme. Once the programme was culturally adapted, remote training was then conducted, whereby local nurses and doctors were trained by UK DESMOND trainers on how to deliver the EXTEND resources.

### Remote training

Training on EXTEND was delivered virtually to six nurses and two doctors, by two National UK DESMOND trainers. Additional meetings were held to ensure that the two doctors were able to carry out quality assurance (QA). This QA method would assess whether the nurses were delivering the programme as intended. Logistical challenges, however, became a barrier to fully allow sessions to be quality assured. In future DSMES delivery, we aim to address these issues, by allocating additional time and resources to deliver a robust QA process.

All six nurses joined the training from one location. This was to guarantee broad band internet connectivity. The nurses did not have access to broad band internet and would have had to rely on mobile data, a service that tends to be unstable and expensive. To overcome these challenges the Principal Investigator (PI) in Ghana made the necessary arrangements and projected the training sessions at the hospital. There were minimal challenges with technology during the training. One doctor was also physically present at the hospital with the nurses. The other doctor joined remotely.

The core aim of the training was to ensure that the clinicians were equipped with skills to facilitate a group-based self-management programme. In particular, the training included key topics around increasing knowledge of the DESMOND philosophy and theories (person empowerment and Social Cognitive Theory [9]) emphasising the importance of a person-centered approach when delivering a self-management programme. These areas formed the foundation of the training, to ensure that there was a clear understanding that group-based programmes like EXTEND are less didactic and more tailored to the person's needs. Furthermore, the virtual training also allowed clinicians to familiarise themselves with core educator skills and behaviours and were provided with the opportunity to ‘have a go’ at delivering the sessions. This approach fitted with the core DESMOND theory for educators to develop their own self-efficacy and self-belief in delivering sessions, by providing them with the opportunity to master a task in a controlled and safe environment (mastery experience) and observing others around them carrying out the facilitation.

Following the completion of the two-day virtual training by the UK based team, the PI held two 40-min follow-up virtual meetings with the nurses to address any outstanding queries from the training and to ensure they felt confident to deliver the sessions to the study participants. The pathophysiology of diabetes formed the chunk of the deliberations.

A final virtual training was held after the nurses had delivered two complete sessions of EXTEND. This session was led by the PI and it was mainly to debrief the nurses and provide feedback on the sessions they had already delivered. It also offered the opportunity to introduce additional pictorial modifications to the EXTEND resources and further deepen the nurses' understanding of the pathophysiology of T2D. They were introduced to the diabetes remission trials [14, 15].

### Adaptations of the EXTEND resources

**Fig. 1:**
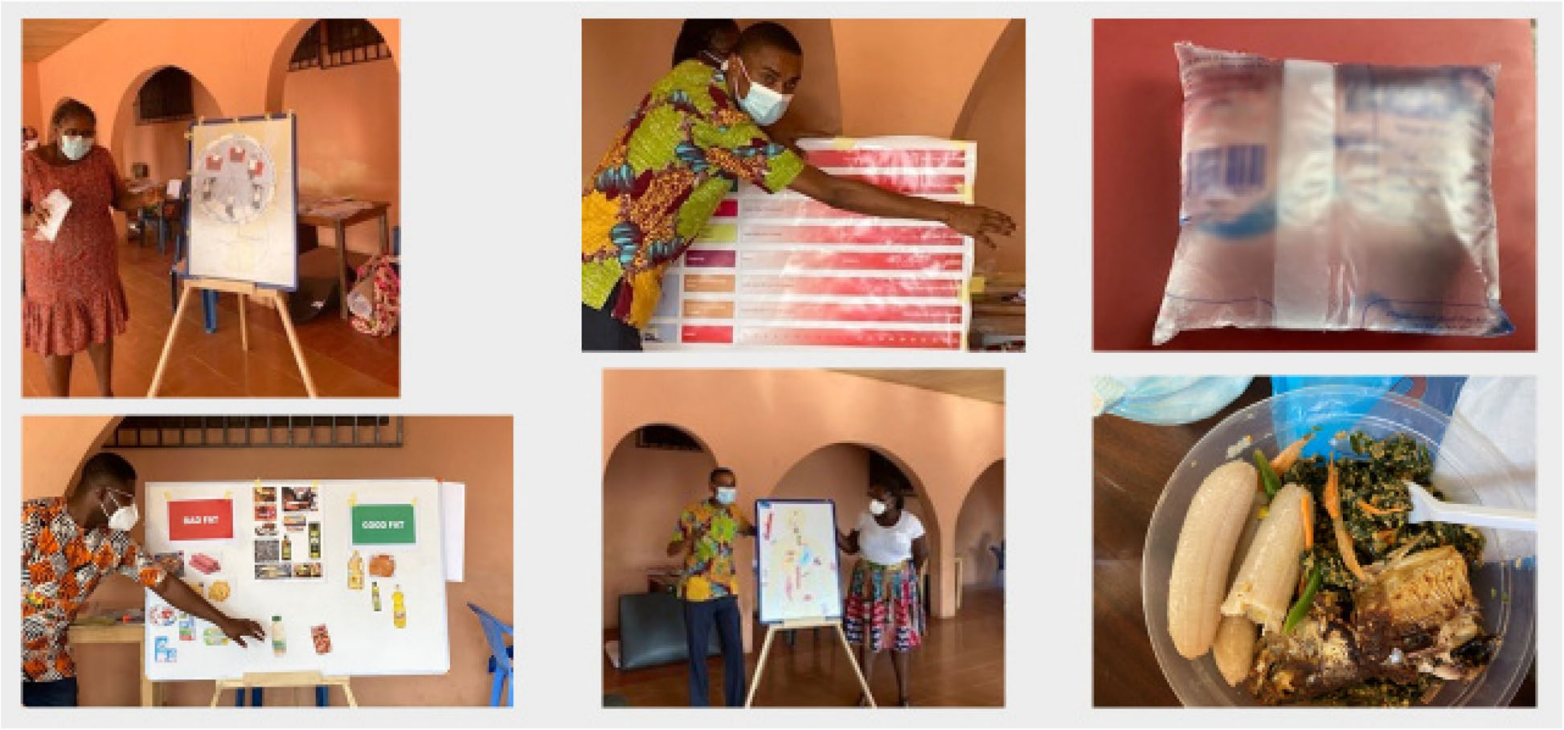
Examples of resources and materials used for the DSMES delivery

The resources and materials shared by the UK partners, were culturally adapted as detailed below. These resources included participant handouts, laminated cards of local food items and pictorial versions of these adaptations (Fig. 1). Organisational and logistical changes were also made to enable a smooth delivery of the DSMES sessions at two sites in Accra: Korle-Bu Teaching Hospitals (KBTH) and Weija Gbawe Municipal Hospital (WGMH). These changes were mainly due to logistical differences across countries (UK and Ghana), but also due to the social distancing rules during the COVID-19 pandemic:

- Adaptation of the set-up of the programme

We considered the option of hiring a school classroom located on KBTH campus. This would mean the sessions could only be held on weekends, holidays, or afternoons, in other words when the school was not in session. We also considered that the majority of people used public transport and were elderly. It was therefore agreed that sessions would be delivered in the mornings. As this programme was part of a wider trial, the use of a venue outside the hospital setting would have raised questions around future implementation. We therefore had to make modifications, which allowed us to deliver the intervention at the study sites.

(b) Delivery logistics

A magnetic board with a human body outline and magnets of vital human organs is a key part of the EXTEND programme to illustrate the breakdown of glucose in the body (Fig. 1). One board was posted by the UK partners to Ghana. To ensure it would be possible to implement EXTEND and ensure replicability outside the research settings, it was imperative to create similar resources to the magnetic board for multi-centre delivery of the programme. Large white boards and tapes were used to relay the key messages about glucose breakdown. For efficient use of time both sides of the board were used, whereby pictures for the subsequent lessons were taped to the reverse side of the board.

The majority of the DESMOND activities are group-based activities that consist of various resources (i.e. food models or images). These types of activities are often presented on a table to generate group discussions and allow people to reflect on their own experiences. Due to the COVID-19 restrictions, we adapted these activities and presented them as large, laminated pictures (Fig. 1). This enabled the facilitators to use the white boards while maintaining social distance.

(c) Adaptation of the delivery of the intervention

DESMOND is a 6-h programme with breaks throughout the day [9]. People who attend clinics, typically arrive very early, often before dawn, to get ahead of the queue, which is something that we experienced with our DSMES programme. We therefore, decided to serve breakfast prior to the start of the sessions and to move the lunch break to the end of the sessions. The intervention was therefore, delivered over about 5 h instead of 6 h, without compromising the key messages of each session. We also decided that the food served to participants would be referred to as examples during the delivery of EXTEND sessions around food choices. Breakfast was a bowl of green salad with avocado, flat bread, and an unsweetened hot cocoa beverage. Lunch was Nkontomire sauce with four fingers of boiled green bananas and water (Fig. 1).

(d) Cultural adaptation of the DSMES content

#### Adaptation of metaphors, analogies and examples

The glucose story as delivered by EXTEND simply states that end-product of ingested carbohydrate is sugar. In Ghana similar to the typical African cuisine, local meals are high in starchy carbohydrates and the typical portions consumed per meal are large [16]. It is not unusual for an average sized person to consume 1 fist sized ball of kenkey in a meal: this being equivalent to about 400 g of carbohydrates.

We were aware from clinical experience that individuals struggled to reconcile the sugar content of their diets with the amount of carbohydrate in the meal. Identifying carbohydrates in a mixed meal is also challenging. Leveraging on our clinical experience we adapted the EXTEND glucose story and placed emphasis on identification of carbohydrates, carbohydrate counting and portion control. We adapted the ‘glucose story’ as follows: The local corn mill, called Nikanika is readily identified by locals. It is operated commercially in markets for grinding staple foods/spices e.g.-corn, beans, soyabean, groundnut, shrimps, and pepper. We likened the process of digestion to the functioning of the Nikanika; explaining that all carbohydrate is broken down to sugar. Although corn is put into the corn mill what comes out of the mill is corn powder. In the same way all carbohydrates (e.g. kenkey, plantain, pawpaw or Nkontomire) would come out as sugar. The amount of sugar produced will depend on the type and amount of carbohydrate consumed.

To describe the four main types of carbohydrate, we used a chest of drawers as an analogy. Carbohydrate examples were the starches, milk and milk products, vegetables, and fruits. Even though each drawer is different, they were all called carbohydrates because the end product of digestion was sugar. We then used the analogy of hats and shoes, to explain that all carbohydrates break down into sugar. For example, even though there are different types of hats to protect us from the sun, all hats are worn the same way on the head. Similarly, all shoes cover the feet and all carbohydrates become sugar. If the item is not worn on the head, it is not called a hat. If the item is not worn on the foot, it is not called a shoe and if the item is not broken down into sugar, then it is not called a carbohydrate. If it is called carbohydrate, then it means that the end-product will include sugar.

Having understood these basics, it suddenly dawned on the participants that they needed to know the sugar content of food. We explained to participants that carbohydrate was counted in units called grams, however, to ensure this was clear across all literacy levels, these were counted in cedis. Our previous experience with people with low health literacy and numeracy skills was that abstract counting was difficult. Yet, dealing in currency was somewhat intuitive. We therefore explained that a cube of sugar was equivalent to five Ghana cedis. We proceeded to provide examples of the amounts of sugar in staple foods e.g. a fist size ball of kenkey equaled to 400 cedis of sugar, a finger of Apentum plantain equaled to 60 cedis of sugar, one soup ladle of nkontomire equaled to 10 cedis of sugar.

#### Adaptation to local foods

As per the DESMOND philosophy, we aimed to share information on food, by using local foods as examples. Nuts are readily available in Ghana, being a tropical country, and therefore, facilitators used nuts as an example to present information around portion control and different types of fats (i.e. saturated, mono-saturated, polyunsaturated). Facilitators also used water as an example to illustrate quantity for oil. In Ghana, water is typically bagged into 500 ml sachets (Fig. 1). Facilitators portioned the sachet into five 100mls portions. Using the 100mls example, facilitators then explained that 100 g of oil was equivalent to 100mls of oil. This visual representation allowed the groups, to reflect on the amount of oil required when cooking. Subsequently, facilitators explained that 100 g of coconut oil had 90 g/90 cedis of saturated fat; palm oil had 50 g/50 cedis of saturated fat and olive oil had 14 g/14 cedis of saturated fat. The simple illustration of the different types of fat, provided better understanding for its use.

## Results and Discussion

### Recommendations for DSMES cultural adaptation in LMICs like Ghana

This paper has described the collaborative work between partners in the UK and Ghana, to develop culturally and linguistically appropriate content to aid the delivery of EXTEND. To our knowledge, this is the first structured DSMES programme adapted to meet the needs of Ghanaians with T2D.

The relevance of diabetes self-management education to long-term diabetes outcomes cannot be over emphasised. In the COVID-19 era and possibly beyond, the need to deliver DSMES in a structured manner in Ghana is glaring. Delivering any self-management education to people with low health literacy is challenging especially when compounded by low numeracy, low literacy and low resource settings. We therefore, sought to build on an existing evidence-based, and theory-guided diabetes self-management programme for the Ghanaian community. To do this, we further adapted the curriculum and resources of an a DSMES programme, a programme that was previously developed and tested in two low- middle- income countries, Malawi and Mozambique [11].

### Limitations

Despite the novelty of this study illustrating the adaptation process of a structured education programme in Ghana, it is important to acknowledge its limitations. The team involved in the adaptation process consisted of clinicians, sharing their expertise from a professional perspective. This process would have benefited a formal consultative process, involving stakeholders and community/lay people, who are key to the development of self-management programmes. Future studies should consider involving individuals from diverse backgrounds (clinical and non-clinical) to enhance the validity of the adapted materials and the generalisability of the programme. This study aimed to adapt a self-management programme for urban settings in Ghana. Further research may be required to ensure that self-management programmes are adapted appropriately for rural areas also.

Although the UK team followed the standard process to train educators, the pandemic added complexities to the delivery of the training. Overall feedback was positive from those trained remotely, however, in-person training would have allowed for an organic interaction between the trainers and educators. The communication barrier was addressed by ensuring that the Ghanaian and UK team held regular virtual calls to resolve any queries ahead of the delivery of the programme.

The ultimate goal from this study, was to test the adapted programme in a randomised controlled trial, which is presented in a separate manuscript. Prior to this step, the adaptation process would have benefitted with the opportunity to test the delivery of the programme in a feasibility study. The nature of this study did not allow for this to take place, however, future studies should consider piloting the programme to refine the adapted material.

### Key recommendations

**Fig. 2:**
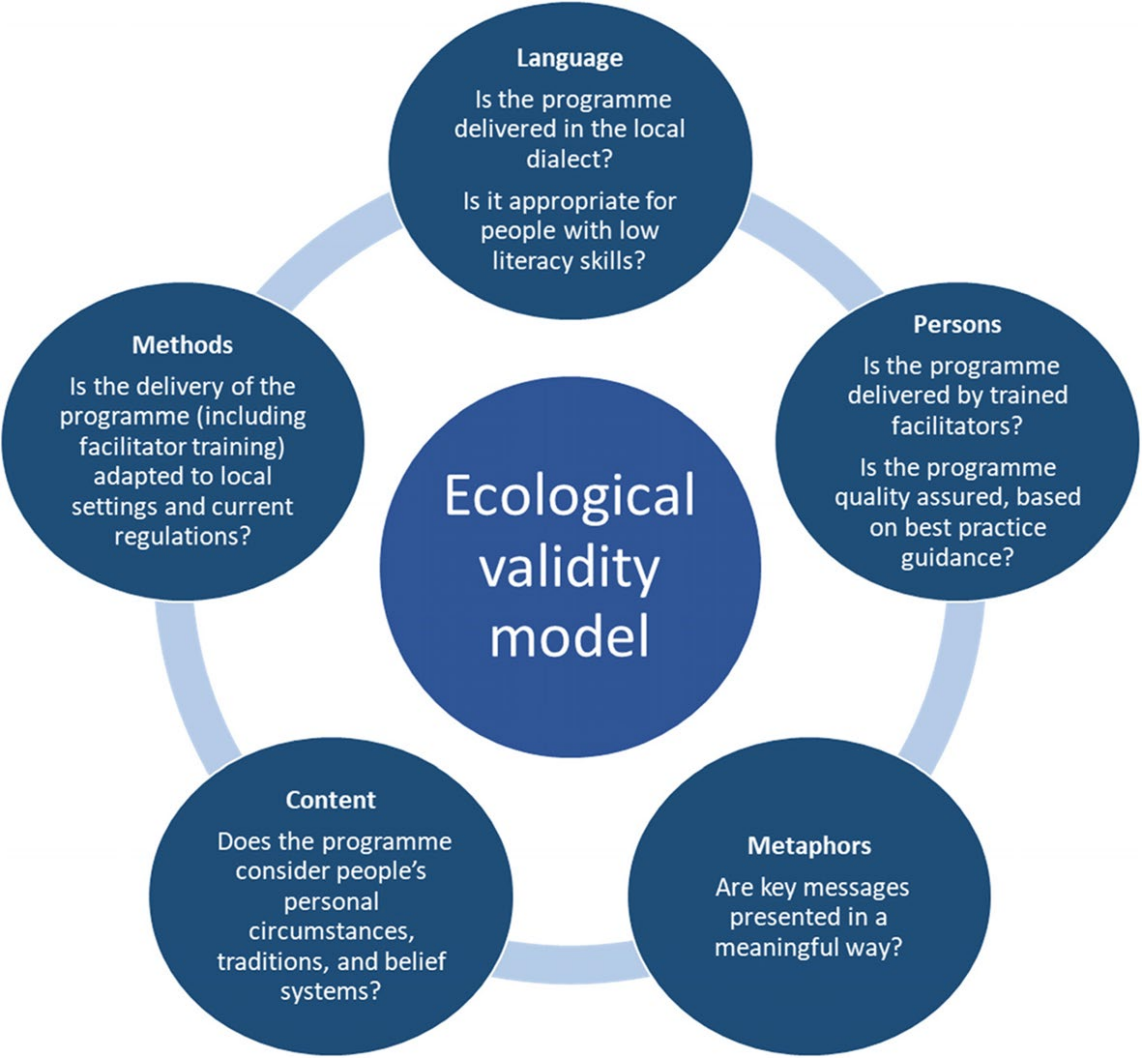
Cultural adaptation of a DMSES programme, key factors to consider based on the Ecological Validity Model

Throughout this development process, we made significant changes and adaptations to the content, logistics and delivery of the programme, tailored to people’s lifestyle needs and demands. Using the five dimensions of the Ecological Validity Model (EVM), a framework designed to guide culturally sensitive adaptation of programmes [17], we summarise key examples of these changes in Table 1; and share recommendations to fellow researchers and stakeholders who wish to culturally adapt DSMES for their communities (see Fig. 2):

(i) Language: Culturally appropriate language is crucial to ensure that information is relayed in a meaningful way. We have since become aware of the community-based participatory research model and the cultural adaptation process [18]. These are key approaches that allow for coherent documentation of the processes involved in adapting a programme thus making it more replicable.
(ii) Persons: For DSMES programmes, it is important to train facilitators who are part of the local communities. Regardless of whether trained facilitators are clinicians or lay people, the training equips facilitators with core facilitation skills to deliver a self-management programme. Such training is built on a philosophy that trained facilitators are not perceived as ‘experts’ to give advice to the group, but rather, to facilitate the group effectively in a person-centered approach.

**Table 1:** Examples of adaptation

	**EXTEND programme**	**Adapted Ghana programme**
**Language**	English, Portuguese and Chichewa	English, Ga, Twi
**People**	Trained educators were nurses and lay people with T2D	Trained educators were nurses and a doctor
	Programme was targeting adults with T2D from Malawi and Mozambique with low literacy levels	Programme was targeting adults with T2D from Ghana with low literacy levels
**Metaphor**	Lock and key analogy	Corn Mill analogy
		Chest of drawers analogy (to illustrate different groups of vegetables-starchy carbohydrate, vegetables, milk and milk products and fruit)
		Hats and shoes (to illustrate that carbohydrates have different amounts of sugars
**Content**	Glucose story (carbohydrates turn into sugar)	Glucose story (carbohyrates are grinded down into sugar)
	Units in grams	Units counted in cedis (1 g equivalent to 1 cedi)
	Quantity of oil using examples of oil	Quantity of oil using examples of water sachets (water in 1 sachet divided into 5. One portion used to represent 100 g of oil)
	Examples of carbohydrate food used to illustrate key messages (i.e. rice pudding, cola, jam, yam raw, sweet potato)	Examples of carbohydrate food used to illustrate key messages (i.e. plantain varieties, kenkey, banku, fufu, yam varieties, rice, wheat, oats, gari, corn, sweet potato, bread)
	Examples of physical activity examples (i.e. cycling, walking, cleaning house)	Examples of physical activity examples (i.e. hill climbing, aerobics, group street jogging “keep fit club” walking, hand washing, sweeping, cleaning house)
	Examples of good/bad fats (i.e. soya, coconut oil)	Comparison of good/bad fats (i.e. olive, palm and coconut oil)
	Diabetes self-management plan	Verbal discussions on concrete next steps and plans regarding diabetes self-management
**Methods**	Magnetic board with a human body outline and magnets of human organs	(when the magnetic board was unavailable) Large white boards with masking tape and marker pens
	Use of food models	Use of laminated images of local dishes
	Attended in the morning, no breakfast offered	Attended in morning, breakfast offered
	Self-management programme delivered in two 3-h sessions by two trained educators	Self-management programme delivered in one 5-h session by two trained educators
	Facilitator training was delivered in person	Facilitator training was delivered virtually

Following best practice guidance and international standards for diabetes self-management education [7, 19] quality assurance must be an integral aspect of programme delivery. This ensures that the fidelity of the programme is maintained. We recommend that initial sessions be kept to the minimum number of 5 participants and to run single sessions instead of concurrent sessions until all logistic challenges have been identified and resolved. Ultimately, standards for quality of structured education programmes must be established by the relevant authorities in each country.

(iii) Metaphors: This is a key dimension to consider when adapting a programme. Metaphors can vary across cultures. For example, a pear in the UK refers to a sweet fruit but in Ghana pears refer to avocados; in the UK a tart refers to pastry with sweet or savoury filling, in Ghana it refers to a bun. To overcome such issues, ensure that any international metaphors are replaced with meaningful explanations that would be relevant and relatable to the local community.
(iv) Content: Consider the traits of the population, including peoples’ personal circumstances and traditions, belief systems, socio-economic and educational level. Be aware that your local community may come from a low socio-economic background, with extremely low literacy and health literacy levels. To ensure that key health messages are delivered in a meaningful way, consider further adaptations where needed to replace written text with visual materials, using examples that are relatable to the priority population. Also, ensure that the programme is delivered at a venue that is easily accessible and is perceived safe for participants.
(v) Methods: Logistics can pose significant setbacks in organising a structured DSMES programme in low resource settings. The planning stage is crucial and it is important to do small trial/test runs prior to the actual roll out of the programme. During a time where the COVID-19 pandemic has interrupted the delivery of face-to-face programmes, it is important to share our learnings that it is possible to deliver DSMES training using live-virtual platforms. However, these virtual sessions must be supplemented by additional virtual sessions delivered by local experts.

## Conclusions

To our knowledge, this is the first adaptation of an evidence- and theory- based structured DSMES programme tailored to the Ghanaian population. The partnership between UK and Ghana has led to the successful adaptation of a DSMES programme. For future implementation of DSMES programmes in LMICs, it is fundamental that such programmes are appropriately adapted to meet the cultural and personal needs of the local communities. We recommend that widely used adaptation models, such as the EVM, are considered for future DSMES adaptation work in LMICs, to ensure that all factors are included and budgeted appropriately. The Ghana DSMES programme has been tested in a randomised controlled trial and the findings will be published in a peer-reviewed journal.

## Data Availability

All data generated or analysed during this study are included in this published article.
